# Inventing an arsenal: adaptive evolution and neofunctionalization of snake venom phospholipase A_2 _genes

**DOI:** 10.1186/1471-2148-7-2

**Published:** 2007-01-18

**Authors:** Vincent J Lynch

**Affiliations:** 1 Department of Ecology and Evolutionary Biology, Yale University, New Haven, CT, 06511, USA

## Abstract

**Background:**

Gene duplication followed by functional divergence has long been hypothesized to be the main source of molecular novelty. Convincing examples of neofunctionalization, however, remain rare. Snake venom phospholipase A_2 _genes are members of large multigene families with many diverse functions, thus they are excellent models to study the emergence of novel functions after gene duplications.

**Results:**

Here, I show that positive Darwinian selection and neofunctionalization is common in snake venom phospholipase A_2 _genes. The pattern of gene duplication and positive selection indicates that adaptive molecular evolution occurs immediately after duplication events as novel functions emerge and continues as gene families diversify and are refined. Surprisingly, adaptive evolution of group-I phospholipases in elapids is also associated with speciation events, suggesting adaptation of the phospholipase arsenal to novel prey species after niche shifts. Mapping the location of sites under positive selection onto the crystal structure of phospholipase A_2 _identified regions evolving under diversifying selection are located on the molecular surface and are likely protein-protein interactions sites essential for toxin functions.

**Conclusion:**

These data show that increases in genomic complexity (through gene duplications) can lead to phenotypic complexity (venom composition) and that positive Darwinian selection is a common evolutionary force in snake venoms. Finally, regions identified under selection on the surface of phospholipase A_2 _enzymes are potential candidate sites for structure based antivenin design.

## Background

Phospholipase A_2_s (PLA_2_; EC 3.1.1.4) are esterolytic enzymes that hydrolyze glycerophospholipids at the *sn*-2 fatty acyl bond, releasing lysophospholipids and fatty acids. PLA_2_s play key roles in various biological processes in mammals including signal transduction, lipid digestion, host defense and production of eicosanoids and other lysophospholipid derivates with potent biological activities [1]. PLA_2 _enzymes are also the major components of snake venoms where they function to immobilize and rapidly kill prey [1]. PLA_2_s from elapid venoms (group-I) are structurally similar to pancreatic secretions while PLA_2_s from viper venoms (group-II) are structurally similar to inflammatory secretions [2]. A third group of PLA_2_s (group-III) have been identified from the venom of bees, jellyfish, scorpions and lizards [2] indicating that PLA_2_s have been recruited into a toxic function multiple times in diverse lineages.

Snake venom PLA_2_s are members of large multigene families with diverse pharmacological activities including neurotoxic, myotoxic, cardiotoxic, anticoagulant and hemolytic effects [2]. These diverse activities evolved from an ancestral nontoxic PLA_2 _by a process of repeated gene duplication followed by functional divergence. PLA_2 _toxicity is independent of enzymatic activity [3,4] and is mediated through "pharmacological sites" on the protein surface that directly interact with ligands on the cell membrane [5]. Thus, the surface of PLA_2_s forms a scaffold for adaptive modification that has been used to generate a diverse array of pharmacological effects through a process of neofunctionalization (the generation of new protein functions that were not the primary function of the ancestral protein).

Previous studies of PLA_2 _genes identified accelerated evolution of group-I genes from *Naja naja *[6] and group-II genes from *Trimeresurus *[7] and *Vipera *[8] consistent with positive Darwinian selection, however these studies focused on one or two species, included relatively few genes and used methods that lack power to detect episodic adaptive evolution. A larger study of group-I and -II genes found that amino acid substitutions were correlated with surface accessibility [9], suggesting that modifications of surface residues and positive selection play important roles in generating toxin diversity. To further explore this possibility I compiled an extensive dataset of full length snake venom PLA_2 _genes from public databases, inferred the gene trees for these toxins and tested for episodes of positive Darwinian selection coincident with the origin of novel pharmacological effects and recurrent diversifying selection on specific sites. In addition, I used a larger amino acids dataset to test if conclusions drawn from the smaller nucleotide dataset were robust to phylogenetic inference.

The results indicate that adaptive evolution is common in snake venom PLA_2 _genes and is associated with the evolution of new toxin functions and speciation events, demonstrating that molecular adaptation has played a pervasive role in the evolution of snakes and their venom arsenal. This analysis has identified the mutational pathway leading from non-toxic to highly toxic PLA_2 _enzymes, reconstructing the processes of mutation and adaptation. Finally, increases in genomic complexity gained through gene duplications has promoted the evolution an increasingly complex phenotype (venom composition), providing a link between molecular, phenotypic and organismal evolution.

## Results and discussion

### Gene duplication and speciation history

**Figure 1:**
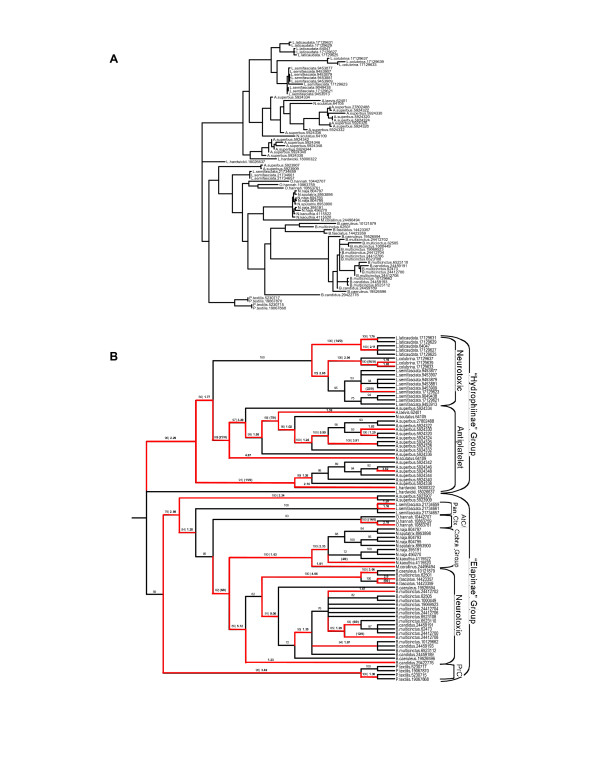
Molecular phylogeny of group-I phospholipase A_2 _genes. (A) Bayesian phylogeny, branch lengths are given as number of substitutions per codon. (B) Evolution of group-I genes. Numbers above the branches are Bayesian posterior probability values (BP) followed by the *d*_*N*_*/d*_*S *_ratio (ω) or the number of nonsynonymous substitutions (N) if no synonymous substitutions (S) occurred along that branch (BP | ω or N/S). Branches in red were inferred to be under positive selection. Genes are labeled according the species they were identified from followed by the GenBank GI number for that gene. Pharmacological effects and higher order classifications are given to the right of clades. Pan, nontoxic pancreatic isoforms. AtC, anticoagulent. Ctx, cardiotoxic. PrC, procoagulent.

**Figure 2:**
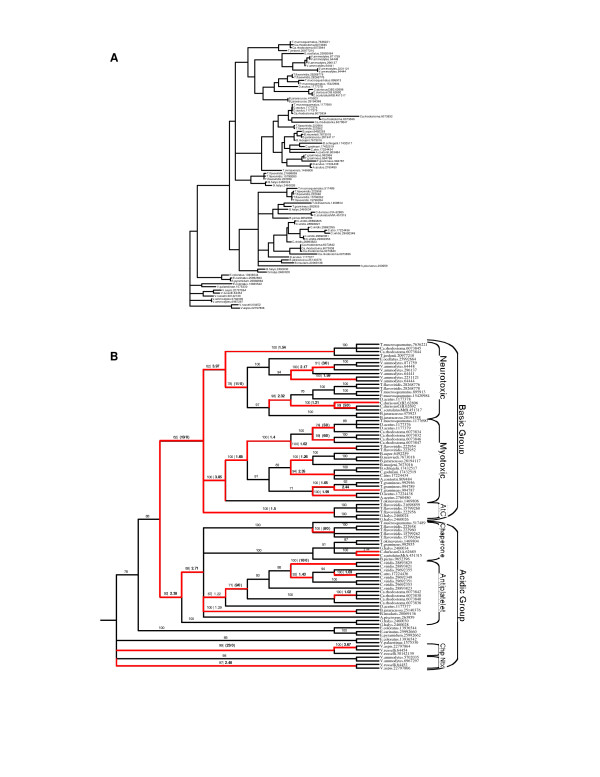
Molecular phylogeny of group-II (B) phospholipase A_2 _genes. (A) Bayesian phylogeny, branch lengths are given as number of substitutions per codon. (B) Evolution of group-II genes. Numbers above the branches are Bayesian posterior probability values (BP) followed by the *d*_*N*_*/d*_*S *_ratio (ω) or the number of nonsynonymous substitutions (N) if no synonymous substitutions (S) occurred along that branch (BP | ω or N/S). Branches in red were inferred to be under positive selection. Genes are labeled according the species they were identified from followed by the GenBank GI number for that gene. Pharmacological effects and higher order classifications are given to the right of clades. AtC, anticoagulent. Chp/Chaperone, neurotoxin chaperone. Ntx, neurotoxic.

**Figure 3:**
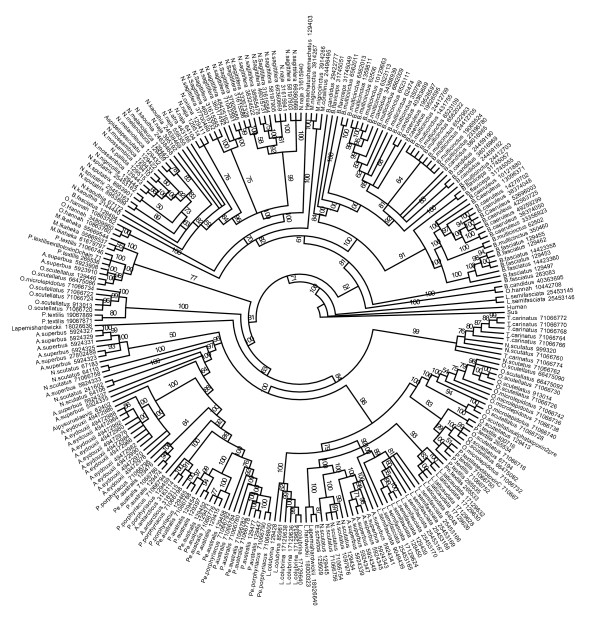
Molecular phylogeny of group-I phospholipase A_2 _genes. Amino acid dataset with Bayesian posterior probabilities shown along branches.

**Figure 4:**
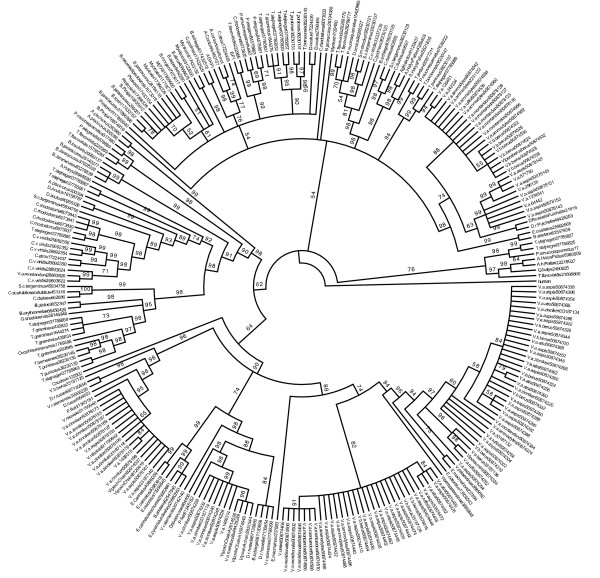
Molecular phylogeny of group-II phospholipase A_2 _genes. Amino acid dataset with Bayesian posterior probabilities shown along branches

To study the molecular evolution of snake venom PLA_2 _genes, I compiled a dataset of 83 group-I and 90 group-II genes from public databases and inferred the evolutionary history of these genes using Bayesian phylogenetics [10]. The molecular phylogeny inferred for group-I PLA_2 _enzymes (Figs. 1 and 3) indicates that genes group with higher-order snake phylogeny and are divided into two sister clades containing marine and Australian species (the "Hydrophiids") and African, American and Asian species (the "Elapids"). This division is similar to the results of earlier phylogenetic studies of group-I PLA_2 _genes [11] and likely reflects a deep divergence between these groups. Within these two major clades, subclades contain genes from closely related taxa that have similar pharmacological effects, suggesting functional diversification occurred after speciation. In contrast, group-II genes cluster by pharmacological effect with little species distinction (Figs. 2 and 4), indicating that functional divergence arose before the divergence of these species.

Even though branch support for the group-I and group-II nucleotide trees was high in this analysis, nucleotide data are only about a third of the gene sequences that are available, the majority are amino acid data (and thus not suitable for codon-based selection analysis discussed later). To test if the topology of nucleotide-based gene trees was sensitive to taxon sampling I inferring group-I and group-II phylogenies using larger amino acids datasets (245 group-I and 271 group-II genes, respectively). Although the trees inferred from amino acid data (Figs. 3 and 4) had lower support for recent lineages than the nucleotide data, perhaps because protein sequences have not accumulated enough phylogenetically informative substitutions to accurately reconstruct recent branching orders, the deeper nodes were well supported and the overall topology was congruent between amino acid and nucleotide data indicating that inferences based on the nucleotide datasets are reliable.

### Origin and elaboration of toxic genes

Snake venom PLA_2_s have diverse pharmacological activities including neurotoxic, myotoxic, cardiotoxic, anticoagulant and hemolytic effects [2], which must have originated after they diverged from nontoxic ancestors. Although uncertainty in the tree topology and the richness of toxin functions makes assessment of specific ancestral and derived functions difficult for all lineages, it is clear from these phylogenies that many novel functions have originated in PLA_2 _genes after gene duplications. For example, three nontoxic group-I PLA_2 _genes isolated from *Laticuadata semifasciata *pancreas [12] branch near the base of the Elapinae group in the nucleotide tree, but are the most basal snake group-I genes in the amino acids tree (Figs. 1 and 3). These pancreatic genes have been suggested to be intermediates between nontoxic and toxic enzymes [12], suggesting that duplication of an ancestral nontoxic gene originally expressed in the pancreas was followed by recruitment into the venom gland and the emergence of toxic functions. Also within group-I, a clade containing neurotoxins from *Laticuadata *has emerged from antiplatelet enzymes; the nested position of this clade indicates that the neurotoxic effect of these enzymes is derived from more ancestral antiplatelet enzymes. The origin of group-II toxins that target muscle is also associated with a gene duplication event. Group-II myotoxins share a unique amino acid substitution at residue 49 (aspartate to lysine) that abolishes enzymatic activity [13,14]. Thus, Lys^49^-myotoxins evolved a novel nonhydrolytic mechanism to induce membrane damage [15,16] after duplication of an ancestral Asp^49^-PLA_2 _that was not myotoxic.

I used maximum likelihood models of coding-sequence evolution [17,18] to test the hypothesis that functional diversification of snake venom PLA_2 _genes is driven by positive Darwinian selection. This method determines the strength and direction of selection by estimating the nonsynonymous-to-synonymous substitution rate (*d*_*N*_*/d*_*S *_= ω), with ω = 1, <1, and >1 indicating neutral evolution, purifying selection and directional selection, respectively. The branch-specific one-ratio model is the simplest, estimating the same ω for all branches in the phylogeny. The estimate of ω for group-I genes under this model, 1.28, is an average over all codons and lineages, highlighting the dominant role of positive selection on elapid venom phospholipases. The estimate of ω for group-II genes under the one-ratio model, 0.686, indicates that group-II genes are generally under purifying selection, however, this estimate is higher than reported from most genes.

The one-ratio model can only detect adaptive evolution when the majority of amino acids and lineages under study have been under positive selection (such as in group-I genes). If adaptive evolution is primarily episodic, then short episodes of positive selection, which are followed by long periods of purifying selection, will not be detected. To test for episodes of positive selection in group-I and group-II gene lineages, I used a free-ratios model that estimates separate *d*_*N*_*/d*_*S *_ratios for all lineages in the tree. These models fit the data significantly better than either the one-ratio model or a constrained one-ratio model with ω forced to be 1 (group-I genes) or a free-ratio model with lineages previously identified with ω >1 constrained to be 1 (group-II genes), indicating that episodes of directional selection are common in snake venom PLA_2 _evolution with nearly 32% and 21% of group-I and -II gene lineages, respectively, having been under directional selection (Figs. 1 and 2). Moreover, there are several branches with extremely high ω values, including two group-I and three group-II branches with ω >3, one group-I branch with ω >5 and one group-I branch with ω = 9.06 (Figs. 1 and 2).

Ohno's model [19] of post-duplication divergence predicts an increase in the nonsynonymous substitution rate following duplication as positive Darwinian selection drives the fixation of mutations that confer new or modified functions on gene duplicates. To test for accelerated evolution after duplication I used smaller datasets for which speciation and duplication events could be unambiguously assigned to each branch and a two-ratios model that estimated different ω parameters for post-duplication (PD) and post-speciation (PS) branches. Surprisingly, in group-I genes PD and PS branches have nearly identical ω values (ω_PD _= 1.12, ω_PS _= 1.22), indicating that positive selection is associated with both gene duplication and speciation. In contrast to group-I genes, group-II gene PD branches evolve much faster than PS branches (ω_PD _= 1.4, ω_PS _= 0.63), consistent with the classical model of neofunctionalization.

Here, neofunctionalization is defined as the emergence of a new toxic effect from an ancestral enzyme that did not posses that effect as its main toxin function (for example, neurotoxic *Laticuadata *genes and Lys^49^-myotoxins discussed above). Strikingly, positive selection occurred in the stem-lineage of 67% (4/6) of group-I functional groups and 88% (7/8) of group-II functional groups (Figs. 1 and 2) indicating that positive selection played a pervasive role in the origin of novel toxin functions during the diversification of vipers and elapids and their venoms. It also suggest lineages which can be targeted for ancestral sequence reconstructions for characterization of ancestral toxin functions to compare extant functions to.

The importance of gene duplication to the evolution of species-specific traits is relatively unknown, but duplications resulting in species-specific adaptations have been demonstrated for some genes [20,21]. The unexpectedly high group-I ω_PS _may be the result of enzyme adaptation to new prey preference after speciation. Indeed, the three semi-aquatic kraits (*Laticaudata sp*.) prey primarily on moray and conger eels and assorted fishes [22,23] while Australian copperheads (*Austrelaps*) prey on frogs and lizards [24]. In the Elapinae group, the king cobra (*Ophiophagus hannah*) and kraits (*Bungarus sp*.) feed almost exclusively on snakes and other reptiles [25], while the true cobras (*Naja sp*.) and the Eastern brown snake (*Pseudonaja textilis*) feed on small mammals, amphibians and birds [25]. This pattern suggest a scenario where dietary shifts after speciation runs the PLA_2 _gene repertoire through a "selective sieve"; those genes which are no longer effective in subduing new prey species are lost, while genes that are still effective adapt to the new prey type and subsequently diversify.

A limitation of the lineage-specific models of protein evolution utilized above is that they can only detect directional selection when the average ω over all amino acids in the protein is >1. Thus, lineage-specific models have limited ability to detect short episodes of directional selection that affect only a few amino acids or amino acids under recurrent diversifying selection. Site-specific models [26] account for rate variation among sites and are powerful tools for detecting diversifying selection. I used three pairs of site-specific models to test for recurrent, diversifying, selection: M0 (one ratio) and M3 (Discrete), M1 (Neutral) and M2 (Selection), and M7 (Beta) and M8 (Beta & ω). Parameter estimates under models M2, M3 and M8, which allow for sites with ω >1, identified that up to 65% of sites in group-I genes and 27% of sites in group-II genes are under positive selection (Tables 1 and 2). This is strong evidence that diversification of snake venom PLA_2 _genes is driven by recurrent positive selection and suggest that venomous snakes are caught in a co-evolutionary arms race with prey as prey evolve resistance to the current venom arsenal and snakes evolve ever more toxic venoms.

**Table 1:** Maximum Likelihood Parameter Estimates for Group-I PLA_2 _Genes.

**Model**	**ℓ**	**ω_0_**	**Parameters**	**Sig.**	**Positive Sites**
Lineage-specific					
M0: one ratio...	-9102.48	**1.28**	**= ω_0_**	P < 0.01	
M0: one ratio-C	-9106.69	**1**	**ω_0 _constrained to 1**		
M0: one ratio-2	-5153.65	1.16	**= ω_0_**		
PD-PS.............	-5153.55		**ω_PD _= 1.22, ω_PS _= 1.12**	n.s.	
Free ratio.........	-8980.48		see Figure 1	P << 0.001	
Site-specific					
M1: neutral.......	-8909.37	0.852	p_0 _= 0.148, ω_0 _= 0		
			p_1 _= 0.582, ω_2 _= 1		
M2: selection...	-8600.95	**2.11**	p_0 _= 0.145, ω_0 _= 0	P << 0.001	36 (PP ≥ 0.99)
			p_1 _= 0.5, ω_2 _= 1		4 (0.95 ≤ PP < 0.99)
			p_2 _= 0.355, **ω_2 _= 4.53**		10 (PP < 0.95)
M3: discrete.....	-8540.48	**1.69**	p_0 _= 0.343, ω_0 _= 0.115	P << 0.001	74 (PP ≥ 0.99)
			p_1 _= 0.435, **ω_2 _= 1.43**		7 (0.95 ≤ PP < 0.99)
			p_2 _= 0.222, **ω_2 _= 4.63**		4 (PP < 0.95)
M7: beta...........	-8725.96	0.594	p = 0.266, q= 0.182		
M8: beta&ω......	-8544.24	**1.4**	p_0 _= 0.70, p = 0.276, q = 0.228	P << 0.001	28 (PP ≥ 0.99)
			p_1 _= 0.297, **ω = 3.41**		2 (0.95 ≤ PP < 0.99)
					9 (PP < 0.95)

L is the log likelihood of the model; ω_0 _is the estimate of the dN/dS ratio under the model (given as a weighted average for the site-specific models); Sig. is the significance of the model when compared to it's neutral partner under the χ^2^-distribution; Positive sites gives the number of sites that fall within a particular Baysean posterior probability value (PP) of being in the site class ω >1. n.s., not significant.

**Table 2:** Maximum Likelihood Parameter Estimates for Group-II PLA_2 _Genes.

**Model**	**ℓ**	ω_0_	**Parameters**	**Sig**	**Positive Sites**
Lineage-specific					
M0: one ratio...	-11770.60	0.686	= ω_0_		
M0: one ratio-2	-4303.29	0.694	= ω_0_		
PD-PS..............	-4300.30		**ω_PD _= 1.41**, ω_PS _= 0.63	P = 0.014	
Free ratios.......	-11580.19		see Figure 1	P << 0.001	
Free ratios-2....	-11591.23	**1**	**ω_0 _constrained to 1**	P << 0.01	
Site-specific					
M1: neutral.......	-11547.79	0.876	p_0 _= 0.124, ω_0 _= 0		
			p_1 _= 0.876, ω_2 _= 1		
M2: selection...	-11266.10	**1.64**	p_0 _= 0.124, ω_0 _= 0	P << 0.001	30 (PP ≥ 0.99)
			p_1 _= 0.605, ω_2 _= 1		2 (0.95 ≤ PP < 0.99)
			p_2 _= 0.271, **ω_2 _= 3.81**		9 (PP < 0.95)
M3: discrete.....	-11096.30	0.971	p_0 _= 0.346, ω_0 _= 0.078	P << 0.001	21 (PP ≥ 0.99)
			p_1 _= 0.42, ω_2 _= 0.83		5 (0.95 ≤ PP < 0.99)
			p_2 _= 0.234, **ω_2 _= 2.55**		8 (PP < 0.95)
M7: beta...........	-11179.33	0.532	p = 0.332, q = 0.293		
M8: beta&ω......	-11073.18	0.899	p_0 _= 0.81, p = 0.352, q = 0.348	P << 0.001	15 (PP ≥ 0.99)
			p_1 _= 0.19, **ω = 2.58**		7 (0.95 ≤ PP < 0.99)
					6 (PP < 0.95)

L is the log likelihood of the model; ω_0 _is the estimate of the dN/dS ratio under the model (given as a weighted average for the site-specific models); Sig. is the significance of the model when compared to it's neutral partner under the χ^2^-distribution; Positive sites gives the number of sites

**Figure 5:**
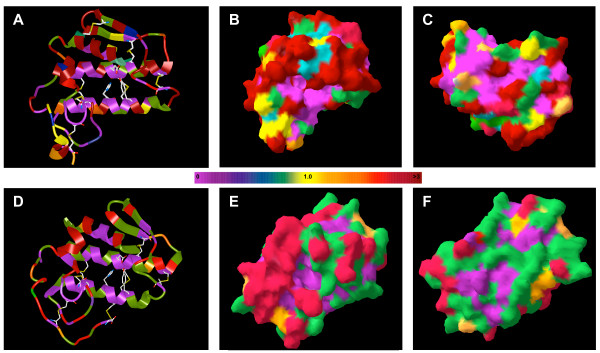
The structure of group-I (A-C) and group-II (D-F) phospholipase A_2 _proteins. The structures are represented by ribbons in A and D with disulfide bonds and catalytic residues shown as sticks and as molecular surfaces rendered in 3D in B, C, E and F. Residues are colored coded according to their approximate posterior mean ω (scale shown between rows) calculated under model M3 (discrete). B and E are in the same orientation as A and D, respectively, while C and F are rotated 180° about a horizontal axis through the molecule.

The three dimensional structure of PLA_2 _enzymes are extremely conserved, obscuring the mechanisms that produce such a wide spectrum of pharmacological effects. To investigate how functional diversity is generated in PLA_2 _enzymes, I mapped sites that were identified as being under diversifying selection on to the crystal structure of group-I and -II phospholipases (Fig. 5). The vast majority of amino acids under diversifying selection occur outside of the α-helicical central scaffold and in regions of the protein that form connecting loops, however, the scaffold of group-II proteins is more conserved than group-I proteins. Functionally important residues, including cysteins that participate in disulfide bonds, the catalytic triad, the calcium-binding site and the hydrophobic channel have *d*_*N*_*/d*_*S *_ratios near zero indicating these regions are under strong structural and functional constraint. In contrast, there are several clusters of amino acids on the molecular surface under intense diversifying selection in both group-I and -II proteins (Fig. 5). These rapidly evolving regions are similar to sites known to produce toxic effects in PLA_2 _enzymes [27-29], highly suggesting that regions under positive selection on the proteins surface are responsible for generating toxic functions.

**Figure 6:**
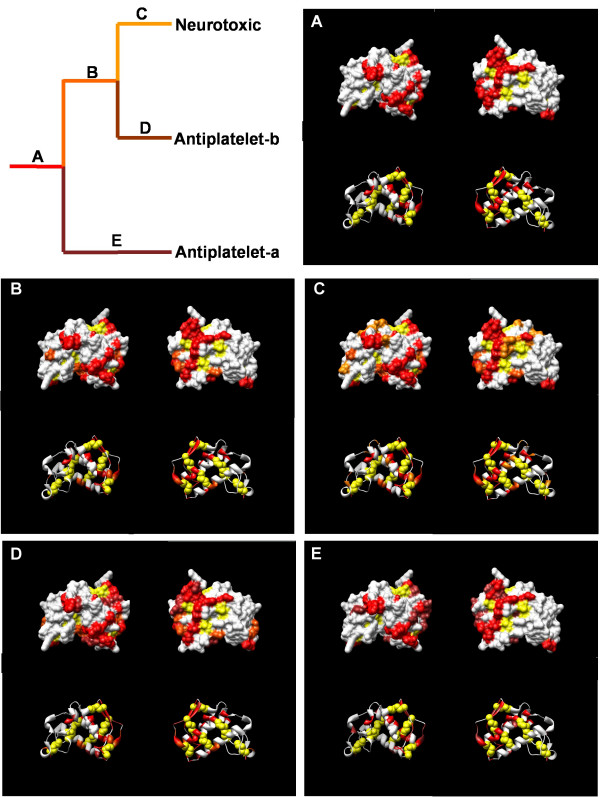
Ancestral sequence mapping for hydrophiinae group genes. The upper left panel shows the generalized phylogeny while each additional panel shows the cumulative amino acid changes that occurred for that lineage. Amino acids in panels A-E are colored by the lineage they changed in. For example, amino acid changes that occurred in the stem-lineage of neurotoxic genes (lineage C, panel C) are colored X; amino acid changes that occurred in ancestral lineages of neutrotoxins are colored Y and Z. In each panel the top two structures are shown with the molecular surface and the bottom structures are ribbons. Structures on left and right are rotated about a central axis 180°. Cystienes are shown in yellow

**Figure 7:**
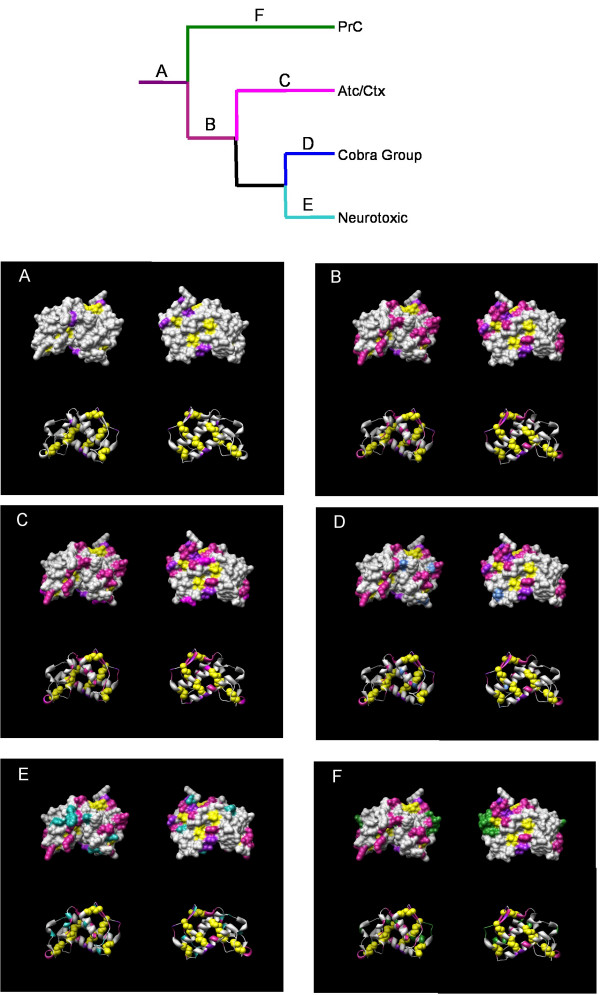
Ancestral sequence mapping for elapinae group genes. Organization follows Figure 6.

**Figure 8:**
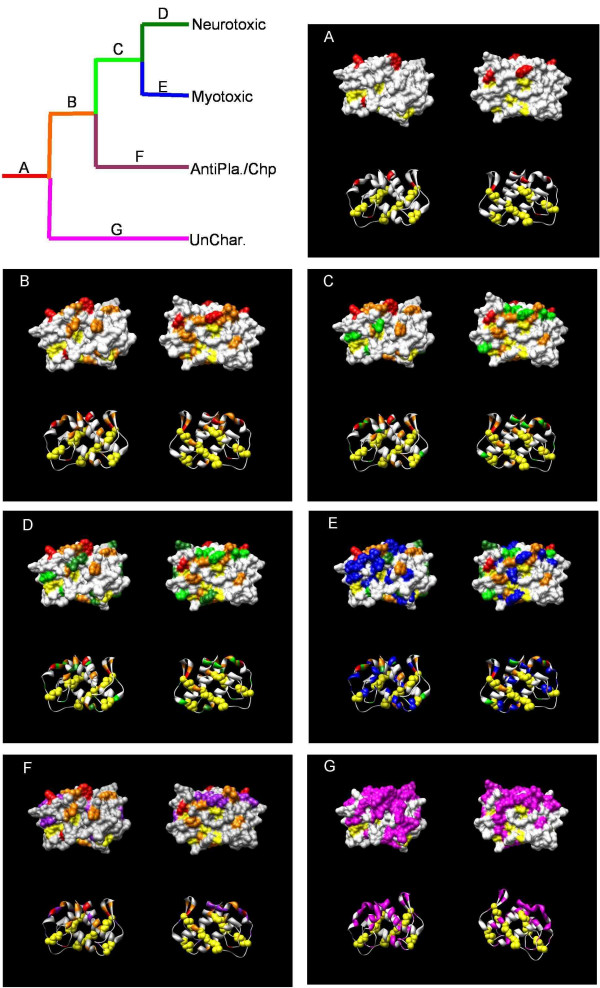
Ancestral sequence mapping for group-II genes. Organization follows Figure 6. Antipla., antiplatelet. Chp., chaperone. UnChar., genes with uncharacterized pharmacological effects.

Although positive selection is often associated with the origin of novel toxin functions (such as antiplatelet, neurotoxic and procoagulent toxins), there are several lineages in which new functions emerge without a significant increase in the nonsynonymous substitution rate (other neurotoxins and cardiotoxins). This is not unexpected since it has long been known that relatively few amino acid changes can have drastic effects on protein functions [ref], suggesting some lineages may have substitutions that contribute to the origins of novel functions but that might have been missed in the lineage and site-specific analyses above. To further clarify the pattern of amino acid replacement that promotes functional changes, I mapped amino acid changes inferred from ancestral sequence reconstructions for select group-I (Figs. 6 and 7) and group-II genes (Fig. 8) onto the crystal structure of these proteins. Clearly, replacements are nearly evenly distributed on protein surface in both group-I and -II genes, but there are several regions that are devoid of amino acids changes including residues in and around the active site and patches of conserved residues on the "back" of the proteins. These regions also contain residues in the slowest evolving site-class from the site-specific analysis. Taken together, these patterns suggest that while only a few changes on the surface are needed to evolve a new function, there are regions under strong structural/functional constraints that limit divergence such as the hydrophobic core and patches of conservation on the surface. Given this, it is interesting to note that several basal clades in the group-II genes with uncharacterized pharmacological effects have strong evidence of selection and many amino acid replacements that map to the surface (Figs. 2 and 8) suggesting that they may have evolved novel, if as of yet unidentified, functions.

## Conclusion

The molecular evolution of group-I and group-II PLA_2 _genes, such as the birth and death like and "selective sieve" processes of gene duplication, divergence and loss are similar to evolution of other snake venom proteins, particularly the elapid three-finger toxins [30]. Indeed, there is even evidence for species-specific toxin adaptation to prey type within the three-finger toxins and maintenance of a well-ordered tertiary structure [30] similar to that seen in PLA_2 _genes, suggesting that this mode of molecular evolution may be common in venom genes.

Kini and Evans [5] have proposed that 'target sites' on the surface of prey cells are recognized by 'pharmacological sites' on PLA_2 _enzymes. These protein-protein interactions determine PLA_2 _specificity by having complementary charges, hydrophobicities, and Van der Waals contact surfaces. This model, combined with the analyses above, suggest that entirely new functions originate after duplication through substitutions in pharmacological sites that alter binding specificities. Although most substitutions will likely disrupt binding specificity for the current target site, a few may create new interaction sites leading to the emergence of novel functions.

The extraordinary level of positive selection acting on snake venom phospholipase A_2 _genes indicates that adaptive molecular evolution plays an important role in the emergence of these novel functions, continues as functions are diversified and refined, and may contribute to niche differentiation after speciation. Interestingly, mapping sites under positive selection onto the structure of PLA_2 _enzymes has identified regions that are attractive candidates for structure-based drug design. These data also demonstrate that increases in genomic complexity gained through gene duplications has lead to an increase in phenotypic complexity (venom composition) and likely the ability of venomous snakes to adapt to new prey types.

## Methods

### Sequence alignment and phylogenetic reconstruction

PLA_2 _genes were obtained from public database (GenBank GI's for each gene are shown in Figs. 1 and 2 for nucleotide data and in Figs. 3 and 4 for amino acid data). Partial sequences, sequences with insertions-deletions that caused reading frame shifts and sequences with premature stop codons were excluded from analysis as likely pseudogenes in the nucleotide analysis. Group-I and -II protein sequences were aligned in Clustal W [31] and adjusted by eye using Se-Al. Bayesian phylogenetic analyses were performed using MrBayes v3.0 [10]. Tree searches were run using four Markov chains for 3,000,000 generations saving every 100^th ^tree and a codon-based GTR+Γ+I model of sequence evolution or a JTT+Γ+I model for amino acid data. Models are nucleotide and amino acid data were selected using ModelGenerator. After stationarity, the final 15000 trees were used to build a consensus tree. Each analysis was performed three times to ensure convergence of tree topologies.

### Tests for selection and ancestral sequence reconstructions

I used codon-based maximum likelihood models implemented in CODEML in the PAML package of programs (version 3.14) [32] to test for lineages under positive selection using the one-ratio and free-ratios models; this package of programs was also used for ancestral sequence reconstructions. To test for differences in post-duplication (PD) and post-speciation (PS) branches I used a smaller dataset of group-I and -II genes that included at least five representatives of each species/pharmacological group and for which gene duplication and speciation events could accurately be assigned for each branch. Twice the log likelihood difference between models, 2Δℓ = 2(ℓ_1_-ℓ_0_), is compared to a χ^2^-distribution with the degrees of freedom equal to the number of parameter differences between the models to test whether the alternative model (free-ratio or PD-PS) fits the data significantly better than the null model (one-ratio). If a lineage has a *d*_*N*_*/d*_*S *_> 1 and the likelihood ratio test is significant, than the neutral model of evolution is violated and positive selection is suggested. To explicitly test for positive selection I used constrained models that fixed ω at 1.

I used three pairs of site-specific models [33] to identify specific amino acids under diversifying selection: M0 and M3 (discrete), M1 (neutral) and M2 (selection), and M7 (beta) and M8 (beta & ω). Model M0 estimates a single ω parameter for all sites and branches, while model M3 (discrete) estimates three independent ω parameters and the proportion of sites belonging to each ω-class directly from the data. Model M1 (neutral) assumes two classes of sites in the protein with the proportion of conserved sites (ω = 0) and neutral sites (ω = 1) estimated from the data, while model M2 (selection) adds a third site class with an additional ω estimated as a free parameter allowing for sites with ω > 1. Model M7 (beta) uses a beta distribution *B*(*p*, *q*) with ω restricted to the interval *(0,1) *while Model M8 (beta & ω) adds a site class with the ω and the proportion of sites with that ω estimated from the data, allowing for sites with ω > 1. Twice the log likelihood difference between the models is compared to the χ^2 ^distribution and tests for variation in ω among sites. After maximum likelihood parameter estimates are calculated, the Bayes theorem is used to calculate the posterior probability of belonging to a site class, when a site is identified with ω > 1 than positive selection is indicated. Sites with posterior probabilities of > 0.5 are reported here. The approximate posterior mean ω for each site from model M3 with two site classes were mapped onto the crystal structures of group-I (PDB ID: 1A3D) and -II (PDB ID: 1OZ6) PLA_2 _proteins using ICM-Browser (available from ) or Chimera. The three-dimensonal space filling structures were generated with Deep View – spdbv v3.7 [34].

## Authors' contributions

VJL designed the study, carried out the statistical analyses and drafted the manuscript. All authors have read and approved the final manuscript.
